# Digestive Ferments

**Published:** 1889-07

**Authors:** 


### DIGESTIVE FERMENTS.

It seems somewhat strange, with our present knowledge of digestive ferments, that the application of pancreatin and pepsin in the diarrhœas and intestinal disorders of children, especially those arising from inanition, is not more general.

We believe that an extension of the use of these products in such diseases would not only prove advantageous to the practitioner, but save the lives of many little ones that otherwise would be doomed.

No practitioner, possessed of a modicum of therapeutic and physiological knowledge, will be found to admit that chalk mixtures, opiates, astringents, etc., meet fairly the indications in these cases. The anti-acids act merely mechanically, soothing the irritated mucous coat of stomach and intestines; the action of opiates, which are especially dangerous to administer to nurslings, is uncertain, for it is impossible to guage their use so as to attain the exact limit essential to intestinal anæsthesia and arrest of peristaltic action, without narcosis; and astringents, while repressing secretion, at the same time retain and favor the absorption of ptomaines and other poisonous products which have provoked the flux—they limit the dejections at the expense of non-elimination of the toxic.

In such cases, and in all those of enfeebled digestion, and in which the food remains undigested and fermenting in the stomach and intestines, pepsin and pancreatin and peptonized foods, afford us pure and simple physiological remedies, whose administration is attended with no dangers; and their employment does not preclude the use of cathartics, or administration of antiseptics that are anti-toxic to ptomaines.

Recently we have obtained the best results from such treatment, though it must be admitted, in cases of unusual gravity, when collapse threatens, that coto and wild yam are sometimes of value to check the flux, the digestive ferment following to secure proper digestion and nutrition. So long ago as 1856, Joulin and Corvisart (Rev. Med. Chir. de Paris) outlined this mode of treatment, and claimed the happiest results therefrom; and more recently it was advocated by Trousseauel, Pidoux, Barthez, and Rilliet, of France, and Ellis and Davidson, of the United Kingdom. Later still, Dr. J. Milner Fothergill (Handbook of Practice, p. 40) remarks of pepsin: "Its utility in the treatment of imperfect digestion, and diarrhœa in children, is certain." Prof. J. Lewis Smith (Prof. Dis. Children, Bellevue Hosp. Med. Coll.—Archiv. Pediatric, 1886, p. 518) expresses exactly the same opinion. Prof. Frederick John Farre (Pariera's Mat. Med. and Therap., p. 943) commends pepsin "very highly in cholera infantum and summer complaints of children." And Bartholow declares (Mat. Med. and Therap-, p. 68): "Very great success has been attained in the treatment of the diarrhœa of infants by pepsin. * * * The motions will be quickly changed in character, and the nutrition of the child improved, by giving it after each supply of food." He further recommends (Naphey's Medical Therapeutics, p. 395) the employment of peptonized milk, or milk gruel for food in these cases, in which he is supported by Wilson Fox (Diseases of Children, ii, p. 821), who considers "pepsin invaluable in gastralgia and all irritative states of intestinal and stomach mucous membranes."

With such evidence, and with the physiological knowledge that at present obtains, it is evident the digestive ferments are too little studied or employed. Yet we must admit there have been good grounds for such neglect, in that the pepsins on the market, for the most part, have been untrustworthy, and with no definite guide for testing, that of the U. S. P. being of a very low standard. These objections no longer obtain, however, for manufacturers have been led to provide accurate tests, and now disseminate the same in their literature. Park, Davis & Co. have issued a work on Digestive Ferments, that is accurate in details, and further, they, and a few other manufacturers, have placed upon the market, a new Pepsin of high digestive power, and possessing the advantage of being absolutely free from ptomaines, and readily soluble. Moreover, the standard of pepsin has been raised by the better manufacturers, and it is the practitioner's own fault if he is not able now to secure a preparation suited to his needs.—Exchange.

**Enforced Circumcision in the Colored Race.**—We call attention to this article in the present number of the JOURNAL. It is contributed by an intelligent colored physician, Dr. Vandavel, and is quite creditable for his first effort at writing for the press. The doctor makes a strong argument in favor of circumcision in his race as a prophylactic measure, and thinks that a long train of evils, besides syphilis, may thus be avoided. It is true, the rhetoric of the article might be improved, the subject more elegantly, perhaps, but not more forcibly put. He makes a plea, also, that the colored people, being ignorant of the laws of hygiene, should be enlightened, while taken under a kind of sanitary protectorate. The paper was read at the annual convention of the Colored Physicians Association.—"Nigger Doctors," as Fisher called them, at Houston; on 25th ult.

---

**Biography of Contemporary Physicians of Texas,** by the editor of this JOURNAL, is being pushed with a vim, and meeting with an enthusiastic response. It will be published as soon as sufficient subscribers are secured, and from the way they are coming in, it will not be long first. Portraits equal to steel engravings will be inserted to a limited number, and fifteen more only will be taken, that of many leading physicians being already secured. No money is required till the manuscript is ready for the press, a certificate to which effect will be issued by disinterested physicians, well and favorably known to all. Write for circulars and terms.

---

**For Sale at a Bargain.**—A good home and practice; a good five room dwelling on a four acre lot, well improved, a good kitchen and dining room, servant's room, and good out houses; fine young orchard and vineyard, good brick cistern; at flourishing railroad depot. Postoffice and express office, also good school.

Address, DR. WM. OSBORNE,
Axtell, McLennan county, Texas.

---

**For Sale.**—A well established practice; a good residence with fine improvements in one of the healthiest ond best towns in the State. For information address editor of this JOURNAL.

